# Rates of cardiovascular events and deaths are associated with advanced stages of HIV-infection: results of the HIV HEART study 7, 5 year follow-up

**DOI:** 10.7448/IAS.17.4.19542

**Published:** 2014-11-02

**Authors:** Stefan Esser, Lewin Eisele, Birte Schwarz, Christina Schulze, Volker Holzendorf, Nobert H Brockmeyer, Martin Hower, Friedhelm Kwirant, Roland Rudolph, Till Neumann, Nico Reinsch

**Affiliations:** 1 Dermatology and Venerology, University Hospital Essen, Essen, Germany; 2 Institute for Medical Informatics, University Hospital Essen, Essen, Germany; 3 Cardiology, University Hospital Essen, Essen, Germany; 4 Clinical Trial Centre Leipzig, University Leipzig, Leipzig, Germany; 5 Department of Dermatology and Venerology, University Hospital Bochum, Bochum, Germany; 6 Internal Medicine, City Hospital Dortmund, Dortmund, Germany; 7 General Practice, HIV-Medicine, Duisburg, Germany; 8 HIV-Medicine, Outpatient-Clinic of Oncology, Essen, Germany

## Abstract

**Introduction:**

Cardiovascular diseases are increasing in aging HIV-positive patients (HIV+). Impact of traditional cardiovascular risk factors, HIV-specific parameters and antiretroviral therapy (ART) on the incidence of cardiovascular events (CVE) and on the mortality rate are investigated in different HIV+ cohorts.

**Methods:**

The HIV HEART (HIVH) study is an ongoing prospective observational cohort study in the German Ruhr area to assess the frequency and clinical course of cardiac disorders in 1481 HIV+ by standardized non-invasive cardiovascular screening. CVE were defined as diagnosed or documented myocardial infarction, coronary heart disease, arterial coronary intervention, stent implantation, bypass operation and stroke.

**Results:**

1481 HIV+ subjects (mean age: 49.3±10.7 years (y), female: 15.6%) were included. 130 CVE and 90 deaths were documented until the end of 7, 5 year follow-up of HIVH. Mean duration of the HIV-infection was 12.9±6.8 y. HIV+ were treated with ART on average for 8.6±6.8 y. According to the CDC classification of the HIV-infection, HIV+ were distributed over the clinical categories (A:34.6%; B:31.4% and C:33.9%) while more than the half had an advanced immunodeficiency (I:8.3%; II:41.1%; III:50.7%). Advanced clinical and immunological stages were significantly (p<0.001) associated with higher incidences of deaths (A:16.7%; B:26.7%; C:56.7% and I:6.7%; II:27.7%; III:65.6%) and CVE (A:17.7%; B:33.1%; C:49.2% and I:3.1%; II:32.3%; III:64.6%) but not with the duration of HIV-infection (per y: Hazard ratio (HR): 0.91 [0.88–0.94]) and ART (per y: HR: 0.81 [0.79–0.84]) adjusted for age. The proportion of deceased HIV+ with HIV-RNA ≥50 copies/mL and lower CD4-cell counts at their last visit is significantly higher compared with living HIV+ without CVE (HIV-RNA ≥50 copies/mL: 25.6% vs 14.7%). Median CD4-cells: 286.5 cells/µL (IQR: 168.8–482.8) versus 574 cells/µL (IQR: 406–786). 96.1% of the living HIV+ with CVE had HIV-RNA<50 copies/mL and median CD4-cells 542.5 cells/µL (IQR: 370–793.5).

**Conclusions:**

Advanced clinical and immunological stages of HIV-infection, but not the duration of ART, were associated with higher incidences of CVE and deaths in the HIVH cohort. These observations support an earlier initiation of ART in HIV+. Special cardiovascular risk calculations for HIV+ should consider immunological and clinical categories of the HIV-infection.

